# In Vitro Thrombogenicity Evaluation of Hemodialyzers

**DOI:** 10.3390/ijms27052164

**Published:** 2026-02-25

**Authors:** Adam M. Zawada, Robert Nitschel, Craig Kamerath, Nathan Crook, Skyler Boyington, Ansgar Erlenkoetter

**Affiliations:** 1 Product Development, Fresenius Medical Care Deutschland GmbH, 66606 Sankt Wendel, Germany; 2 Biosciences, Fresenius Medical Care Deutschland GmbH, 66606 Sankt Wendel, Germany; robert.nitschel@freseniusmedicalcare.com (R.N.); ansgar.erlenkoetter@freseniusmedicalcare.com (A.E.); 3 Applications Laboratory, Fresenius Medical Care, Ogden, UT 84404, USAnathan.crook1@freseniusmedicalcare.com (N.C.); skyler.boyington@freseniusmedicalcare.com (S.B.)

**Keywords:** thrombogenicity, platelet activation, coagulation, dialyzer membrane

## Abstract

Investigation of dialyzer thrombogenicity is a critical step during the development of a new dialyzer. Novel dialyzer membranes aim to reduce the inherent thrombogenic potential of artificial surfaces by, e.g., increasing membrane hydrophilicity. Reliable in vitro testing is fundamental during dialyzer development and must be in line with the current standards. Using the novel FX CorAL dialyzer with its increased membrane hydrophilicity as an example, this study characterizes dialyzer thrombogenicity in an in vitro test setup in line with ISO 10993-4 and identifies factors which influence dialyzer thrombogenicity. In a recirculation setup with human blood, platelet activation (platelet counts, β-thromboglobulin, platelet adsorption), coagulation (thrombin–antithrombin III complex) and complement activation (sC5b-9) were investigated among polysulfone- (FX CorAL, FX CorDiax, Optiflux, xevonta), polyethersulfone- (ELISIO, Revaclear, Theranova) and AN69 ST-based (Nephral) dialyzers. Additionally, the impact of dialysate and electrolyte composition on thrombogenicity was investigated. The FX CorAL showed the lowest platelet activation compared to all poly(ether)sulfone-based dialyzers and lower complement activation compared to most poly(ether)sulfone-based dialyzers and to the Nephral dialyzer. No significant differences were observed between the investigated dialyzers with regard to plasmatic coagulation. Among the tested parameters, the dialyzer showed the strongest impact on the thrombogenicity results. This study proposes guidance on in vitro testing of dialyzer thrombogenicity in line with current standards and may contribute to reducing the current heterogeneity among in vitro hemocompatibility testing.

## 1. Introduction

In vitro hemocompatibility testing is a fundamental part and regulatory requirement during the development of a new dialyzer. During hemocompatibility testing, dialyzer thrombogenicity has to be investigated thoroughly, given the thrombogenic potential of dialyzer materials and the subsequent clotting risk during dialysis treatments [1,2,3,4,5,6]. Guideline documents, such as ISO-10993-4 (Biological evaluation of medical devices—Part 4: Selection of tests for interactions with blood), recommend the evaluation of distinct markers and methods for thrombogenicity testing, including coagulation, platelet activation, complement system and hematology [7]. However, as such guidelines are not specific for dialyzers, the respective methods are not standardized and manufacturers have to pre-define the test conditions and hemocompatibility markers by themselves, leading to a heterogenous landscape of dialyzer hemocompatibility testing [8,9]. Such models may differ in important parameters, such as the origin of blood, storage time of blood, anticoagulation type and amount, setup type (dynamic or static), treatment time, temperature, investigated markers, blood flow conditions, use of dialysate and analysis of data (e.g., timepoints or normalization).

A new dialyzer entering the market shall not lead to a higher thrombogenicity risk than the currently available dialyzers. The two main drivers of thrombogenesis are platelet activation and plasmatic coagulation [10,11,12] and need to be extensively evaluated during in vitro hemocompatibility testing.

In normal hemostasis, platelets are sensors of vascular integrity and get activated after vascular injury to promote thrombus formation and thus prevent loss of blood [10,13,14,15]. However, this process of platelet activation is also initiated during dialysis treatment, due to the contact of blood with artificial surfaces. Dialyzer membranes rapidly absorb plasma proteins and platelets subsequently interact and adhere to the membrane, leading to a reduction in platelets in the circulating blood [8,16,17,18,19,20]. Accordingly, for platelet activation, ISO-10993-4 recommends the measurements of platelet counts (percentage of platelet loss) and specific markers of platelet activation (e.g., β-thromboglobulin [β-TG], platelet factor 4 [PF4], thromboxane B2 [TXB2]) or the characterization of platelet morphology with scanning electron microscopy (SEM) [7].

Besides platelet activation, plasmatic coagulation is the second major pathway of thrombogenesis [10,11,12]. The coagulation cascade involves many plasma factors, including factor Xa or thrombin; the latter promotes the conversion of fibrinogen to fibrin, a glue-like substance and the major end product of the coagulation cascade. To investigate coagulation, ISO-10993-4 recommends the characterization of thrombin generation (via, e.g., thrombin–antithrombin III complex [TAT], prothrombin fragment 1.2 [F1.2]), fibrin (e.g., fibrinopeptide A [FPA]) or the investigation of partial thromboplastin time (PTT) [7].

Modern dialyzers aim to reduce thrombogenicity risk, mainly by improving the membrane composition and geometry, given that the membrane, with its large surface area, is the major point of interaction with the patient’s blood. Currently, most synthetic dialyzer membranes are based on the hydrophobic polysulfone or polyethersulfone and are blended with polyvinylpyrrolidone (PVP) to increase the hydrophilicity of the membrane. Increased hydrophilicity on the blood-side surface of the membrane leads to reduced protein fouling via the repulsive hydration force by the formed water layer, and subsequently improves hemocompatibility [16,17,21,22,23,24,25,26,27].

In the current study, we present an in vitro setup for the characterization of dialyzer thrombogenicity among different commercial dialyzers, including the novel FX CorAL dialyzer with increased PVP content on the blood-side surface. This study aims to provide guidance for dialyzer hemocompatibility testing that is in line with the ISO-10993-4.

## 2. Results

The following sections investigate platelet activation (Section 2.1), plasmatic coagulation (Section 2.2) and complement activation (Section 2.3) in a recirculation setup with human blood. Additional clotting-related control parameters are presented in the subsequent section (Section 2.4), followed by comparison experiments of saline vs. dialysis solutions as well as electrolyte supplementation for the priming and filling of the dialysate side (Section 2.5).

### 2.1. Investigation of Platelet Activation

#### 2.1.1. Determination of Platelet Loss

**Figure 1:**
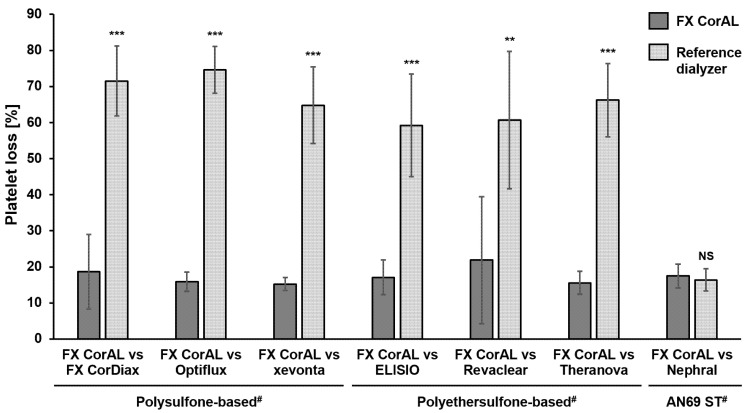
Platelet loss in recirculation experiments with the FX CorAL vs. reference dialyzers. Platelet loss was determined in a blood recirculation setup in pairwise comparisons of the FX CorAL (test dialyzer) vs. seven different reference dialyzers. Means ± standard deviations of six independent experiments are shown for each comparison. The reported level of significance is given with respect to the FX CorAL dialyzer in the respective comparison (** *p* < 0.01; *** *p* < 0.001). ^#^ Membrane material information is provided for the respective reference dialyzers; FX CorAL: polysulfone-based. NS: Not significant.

Platelet loss was determined in the recirculation experiment in pairwise comparisons of the FX CorAL (test dialyzer; Fresenius Medical Care, Bad Homburg, Germany) vs. the other polysulfone- (FX CorDiax [Fresenius Medical Care], Optiflux [Fresenius Medical Care], xevonta [B.Braun, Melsungen, Germany]), polyethersulfone- (ELISIO [Nipro, Osaka, Japan], Revaclear [Baxter/Vantive, Deerfield, US], Theranova [Baxter/Vantive]) and AN69 ST- (Nephral [Baxter/Vantive]) based reference dialyzers. The results of these pairwise comparisons are summarized in Figure 1.

The mean platelet loss across all experiments was in a range between 15.2% (FX CorAL) and 74.6% (Optiflux). FX CorAL showed a significantly lower platelet loss compared to all other polysulfone- and polyethersulfone-based dialyzers. The AN69 ST-based Nephral had a comparable platelet loss (16.4%) to the FX CorAL dialyzer.

#### 2.1.2. Determination of the Platelet Activation Marker β-Thromboglobulin

**Figure 2:**
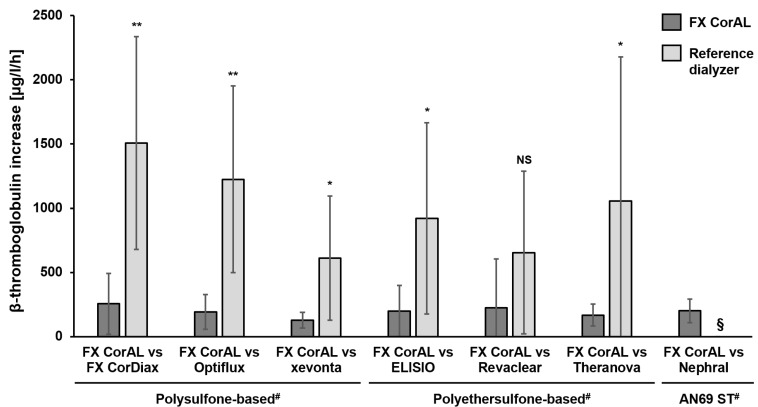
β-thromboglobulin (β-TG) increase over time in recirculation experiments with the FX CorAL vs. reference dialyzers. The β-TG increase per hour was determined in a blood recirculation setup in pairwise comparisons of the FX CorAL (test dialyzer) vs. seven different reference dialyzers. Means ± standard deviations of six independent experiments are shown for each comparison. Standard deviations are presented up to the x-axis, in case of values below 0. The reported level of significance is given with respect to the FX CorAL dialyzer in the respective comparison (* *p* < 0.05, ** *p* < 0.01). ^#^ Membrane material information is provided for the respective reference dialyzers; FX CorAL: polysulfone-based. § For Nephral, no valid signal over time could be observed; this marker could not be analyzed. NS: Not significant.

To further characterize platelet activation by the different dialyzers, β-thromboglobulin (β-TG) was determined in the same recirculation setup. The results are summarized in Figure 2.

The mean β-TG increase per hour ranged from 128 µg/L/h (FX CorAL) to 1507 µg/L/h (FX CorDiax) across all experiments. In line with the platelet loss results, FX CorAL showed the lowest β-TG increase over time, across all polysulfone- and polyethersulfone-based dialyzers. The difference vs. Revaclear did not reach statistical significance due to the high standard deviation. The data for Nephral showed negative results, indicating strong adsorptive characteristics by the AN69 ST membrane. Therefore, these values were not valid for analysis.

#### 2.1.3. Determination of Platelet Adsorption by Fluorescence Microscopy

**Figure 3:**
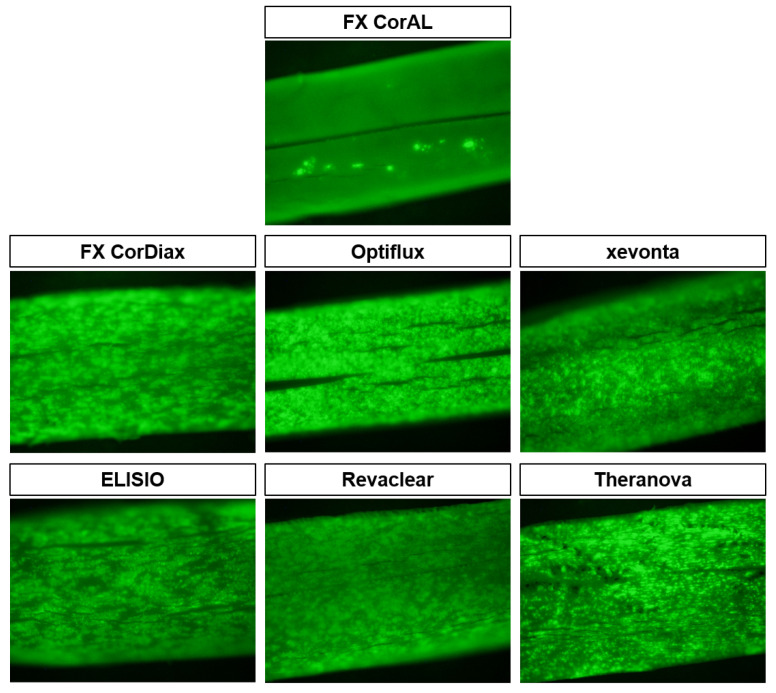
Visualization of adhered platelets by fluorescence microscopy. After blood recirculation, fibers were opened and stained with CD41a-FITC overnight. Platelets were visualized using a BX31 microscope equipped with a DP73 camera and appear as bright green objects. The light green background originates from fiber autofluorescence.

To visualize platelet adsorption by the different membranes after blood recirculation, fluorescence microscopy was performed. Therefore, the adherent platelets were stained with Fluorescein isothiocyanate (FITC)-labeled antibodies targeting CD41a (glycoprotein IIb/IIIa complex). Representative images of open fibers after staining are shown in Figure 3. In contrast to the polysulfone- and polyethersulfone-based fibers, the AN69 ST fibers could not be prepared for staining due to technical issues; therefore, no fluorescence microscopy was performed for the Nephral dialyzer.

In line with the previous results regarding the platelet loss (Section 2.1.1.) and β-TG increase over time (Section 2.1.2.), the images visually confirmed that the FX CorAL showed the lowest platelet adsorption to the membrane. While all other tested membranes showed substantial platelet adsorption across the entire membranes, minimal platelet clusters were observed on the FX CorAL membrane.

### 2.2. Investigation of Plasmatic Coagulation

**Figure 4:**
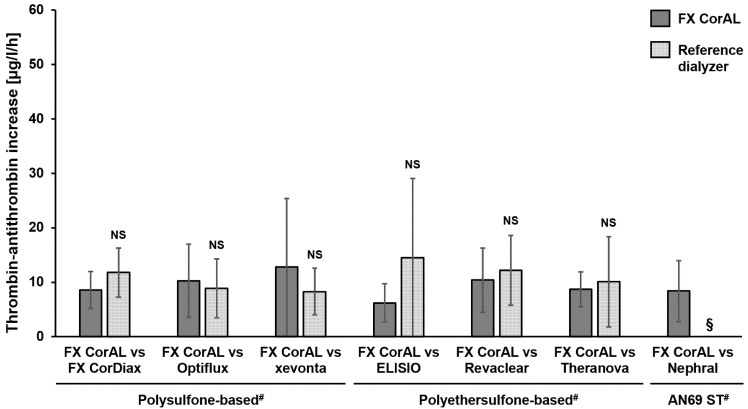
Thrombin–antithrombin III complex (TAT) increase over time in recirculation experiments with the FX CorAL vs. reference dialyzers. TAT increase per hour was determined in a blood recirculation setup in pairwise comparisons of the FX CorAL (test dialyzer) vs. seven different reference dialyzers. Means ± standard deviations of six independent experiments are shown for each comparison. Standard deviations are presented up to the x-axis, in case of values below 0. The reported level of significance is given with respect to the FX CorAL dialyzer in the respective comparison. ^#^ Membrane material information is provided for the respective reference dialyzers; FX CorAL: polysulfone-based. § For Nephral, no valid signal over time could be observed; this marker could not be analyzed. NS: Not significant.

Thrombin–antithrombin III complex (TAT) was measured in the blood recirculation setup to characterize plasmatic coagulation. The results are summarized in Figure 4.

The mean TAT increase per hour was in a comparable range between all polysulfone- and polyethersulfone-based dialyzers, with no significant differences (6.2 µg/L/h [FX CorAL]–14.5 µg/L/h [ELISIO]). Similar to the β-TG determination (Section 2.1.2), the Nephral data could not be analyzed, as no valid signal was measured.

### 2.3. Investigation of Complement Activation

**Figure 5:**
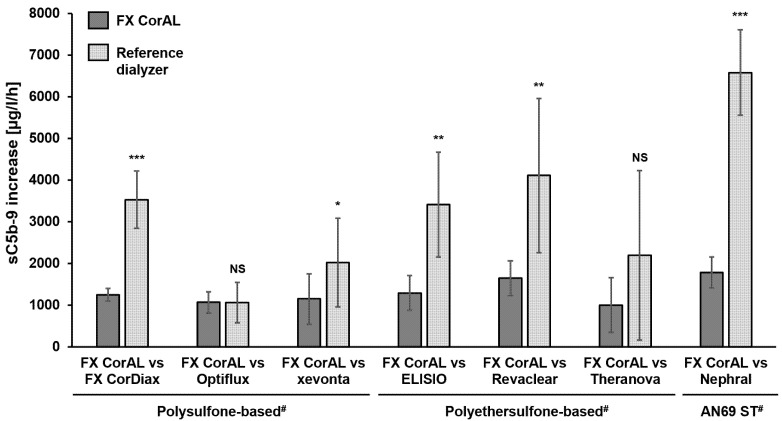
Complement factor sC5b-9 increase over time in recirculation experiments with the FX CorAL vs. reference dialyzers. The sC5b-9 increase per hour was determined in a blood recirculation setup in pairwise comparisons of the FX CorAL (test dialyzer) vs. seven different reference dialyzers. Means ± standard deviations of six independent experiments are shown for each comparison. The reported level of significance is given with respect to the FX CorAL dialyzer in the respective comparison (* *p* < 0.05, ** *p* < 0.01, *** *p* < 0.001). ^#^ Membrane material information is provided for the respective reference dialyzers; FX CorAL: polysulfone-based. NS: Not significant.

According to ISO-10993-4, complement activation was investigated across all tested dialyzers in the blood recirculation setup. The results for the complement factor sC5b-9 are shown in Figure 5.

The mean sC5b-9 increase per hour ranged from 1008 µg/L/h (FX CorAL) to 6585 µg/L/h (Nephral) across all experiments. The FX CorAL dialyzer showed significantly lower complement activation compared to all reference dialyzers, except for Optiflux and Theranova.

### 2.4. Investigation of Additional Clotting-Related Parameters

Besides the investigated parameters recommended by the ISO-10993-4, we additionally determined further parameters which are associated with thrombogenicity, and which help to further assess the in vitro thrombogenicity.

#### 2.4.1. Determination of Activated Clotting Time (ACT)

**Figure 6:**
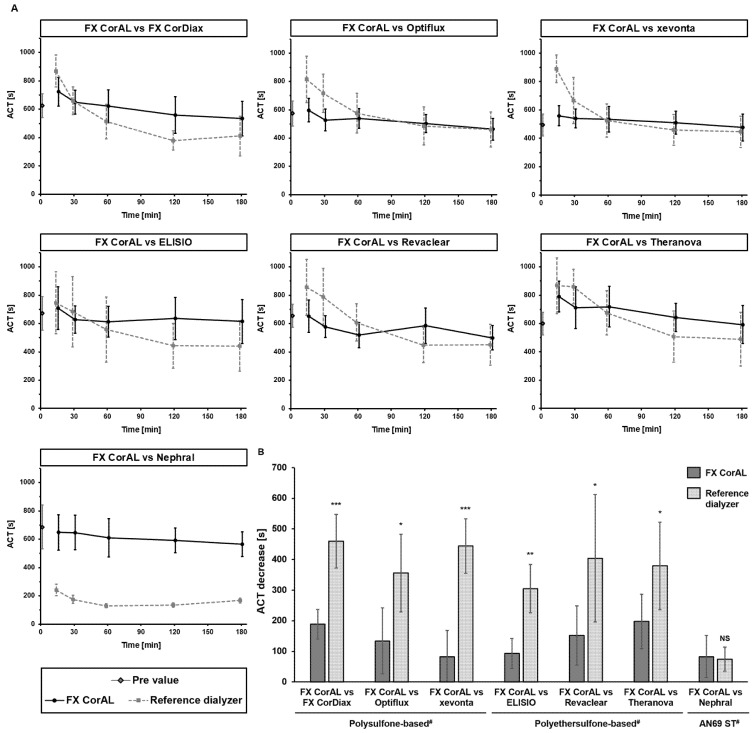
Activated clotting time (ACT) over time in recirculation experiments with the FX CorAL vs. reference dialyzers. (**A**) The ACT was determined in pre-recirculation blood samples (time point 0 min) as well as over time in a blood recirculation setup in pairwise comparisons of the FX CorAL (test dialyzer) vs. seven different reference dialyzers. Means ± standard deviations of six independent experiments are shown for each comparison. For better visualization, the time points are shifted by +/−1 min to prevent overlapping within the figure. The respective timepoints were 0 (pre values), 15, 30, 60, 120 and 180 min. (**B**) The ACT decrease between 15 and 180 min is displayed for each comparison. The reported level of significance is given with respect to the FX CorAL dialyzer in the respective comparison (* *p* < 0.05, ** *p* < 0.01, *** *p* < 0.001). ^#^ Membrane material information is provided for the respective reference dialyzers; FX CorAL: polysulfone-based. NS: Not significant.

The clotting time may be influenced by many parameters that are affected by the dialyzer membrane, such as heparin activity, platelet counts, or plasmatic coagulation activation. Moreover, non-membrane related factors such as dilution effects may also impact the clotting time. Therefore, the ACT may provide additional information for the characterization of dialyzers in the in vitro test setup. The respective results are summarized in Figure 6.

For most dialyzers, ACT increased at the first measurement time point of recirculation (15 min) compared to the pre value (0 min), which can be mainly attributed to the dilution effects at the recirculation start. Compared to all other polysulfone- and polyethersulfone-based dialyzers, the FX CorAL dialyzer had the lowest ACT increase at 15 min, in line with the lowest platelet loss (Figure 1) and the implications of platelets in the coagulation process [28]. After 15 min, all dialyzers showed a reduction in the ACT, corresponding to the ongoing coagulation process during continuous recirculation. The FX CorAL dialyzer showed the significantly lowest ACT decrease compared to all other polysulfone- and polyethersulfone-based dialyzers. The Nephral dialyzer had a completely different pattern compared to all the other dialyzers. The ACT strongly dropped after 15 min and remained at a very low level, in line with the strong heparin binding affinity that the AN69 ST membrane is known for [4,29].

#### 2.4.2. Determination of Ionized Calcium

**Figure 7:**
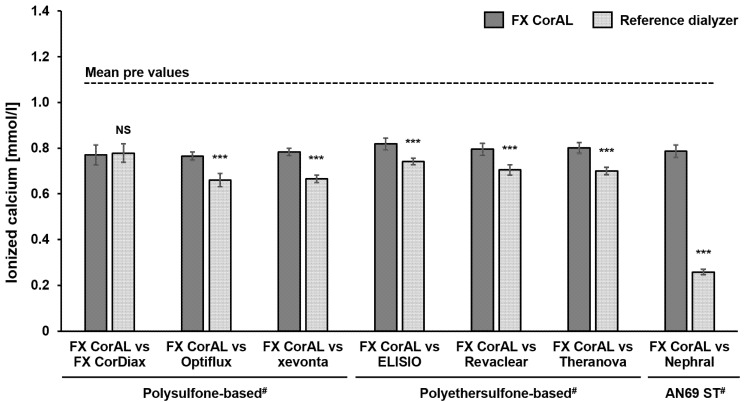
Mean concentration of ionized calcium in recirculation experiments with the FX CorAL vs. reference dialyzers. The concentration of ionized calcium was determined in the blood pool (pre values, 0 min) and at different time points of blood recirculation (15, 30, 60, 120 and 180 min). The mean pre values are indicated by the dashed line and the columns represent the mean ionized calcium concentration across the five sampling time points during recirculation. Means ± standard deviations of six independent experiments are shown for each comparison. The reported level of significance is given with respect to the FX CorAL dialyzer in the respective comparison (*** *p* < 0.001). ^#^ Membrane material information is provided for the respective reference dialyzers; FX CorAL: polysulfone-based. NS: Not significant.

Although the ACT was very low for the Nephral dialyzer, no signs of clotting were observed. Therefore, we additionally determined the ionized calcium levels during blood recirculation with the different dialyzers, given that calcium plays a crucial role in the coagulation cascade. The results for all dialyzers are summarized in Figure 7.

In all recirculation setups, ionized calcium concentrations dropped until the first measurement time point (15 min) and remained constant afterwards (mean pre value: 1.08 ± 0.02 mmol/L; mean value during recirculation across all dialyzers and time points: 0.72 ± 0.14 mmol/L). The Nephral dialyzer showed the strongest drop in ionized calcium concentration due to its strong adsorption behavior (0.26 ± 0.01 mmol/L). The other differences between the dialyzers are rather attributable to the different dialyzer designs leading to different dilution and diffusion effects, due to the different blood and dialysate compartment volumes. The FX CorAL and FX CorDiax have the same dialyzer housing design, and thus there are no differences in the ionized calcium levels.

### 2.5. Investigation of Dialysate vs. Saline Solutions and Electrolyte Supplementation

Given the strong impact of the membrane material on electrolyte composition and the subsequent effect on thrombogenicity, we additionally investigated whether the modulation of electrolytes in the recirculation setup may also influence the thrombogenicity parameters. Therefore, two additional experiments were conducted: (1) the usage of dialysate instead of saline (Section 2.5.1), and (2) experiments with calcium and magnesium supplementation (Section 2.5.2) for priming.

#### 2.5.1. Investigation of Thrombogenicity Parameters Using Dialysate vs. Saline Solutions

**Figure 8:**
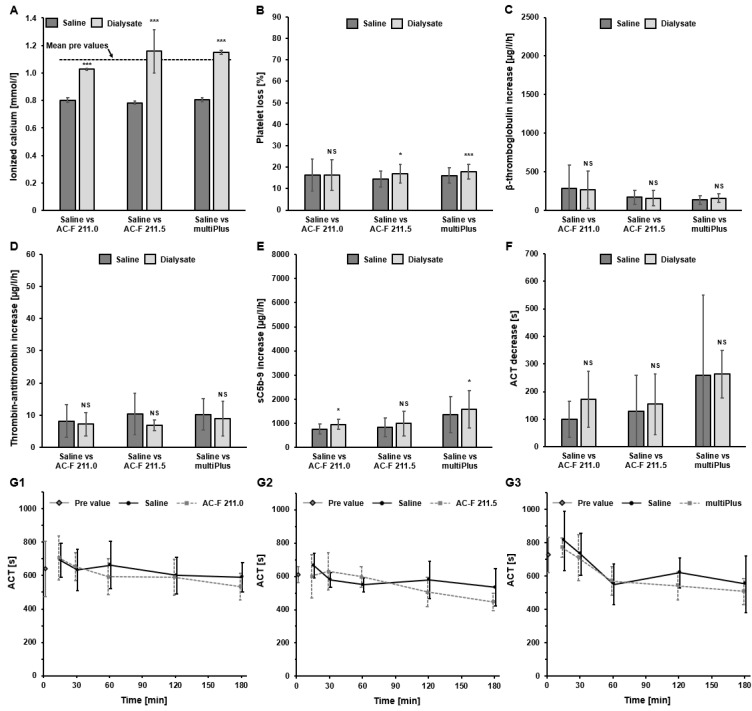
Investigation of thrombogenicity parameters in recirculation experiments with the FX CorAL dialyzer comparing saline vs. dialysate solutions (AC-F 211.0, AC-F 211.5, multiPlus). (**A**) Mean concentration of ionized calcium. (**B**) Mean platelet loss. (**C**) Mean β-thromboglobulin (β-TG) increase over time. (**D**) Mean thrombin–antithrombin III complex (TAT) increase over time. (**E**) Mean complement factor sC5b-9 increase over time. (**F**) Mean activated clotting time (ACT) decrease between 15 and 180 min. (**G1**–**G3**) ACT over time for saline vs. AC-F 211.0 (**G1**), saline vs. AC-F 211.5 (**G2**) and saline vs. multiPlus (**G3**). Measurements and data presentations are according to Figure 1, Figure 2, Figure 3, Figure 4, Figure 5, Figure 6 and Figure 7, respectively. * *p* < 0.05, *** *p* < 0.001, NS: Not significant.

To investigate the potential impact of using dialysate instead of saline as a priming solution on the thrombogenicity parameters, three different dialysate solutions were compared vs. saline in the recirculation setup with the FX CorAL dialyzer (AC-F 211.0, AC-F 211.5 and multi*Plus*). These three dialysate solutions were selected as they differ in their calcium (AC-F 211.0: 1.00 mmol/L, AC-F 211.5: 1.50 mmol/L, multi*Plus*: 1.50 mmol/L) and/or magnesium (AC-F 211.0: 0.50 mmol/L, AC-F 211.5: 0.50 mmol/L, multi*Plus*: 0.75 mmol/L) concentrations (complete composition of the three dialysate solutions is given in the Section 4). The results for all parameters are summarized in Figure 8.

As expected, the calcium concentrations in the setups with dialysate solutions were significantly higher compared to the setup with saline, especially in the two setups with a calcium concentration of 1.50 mmol/L (AC-F 211.5 and multi*Plus*) (Figure 8A). When comparing the impact of using dialysate instead of saline on the investigated thrombogenicity parameters, no significant differences were observed for β-TG, TAT, and ACT (Figure 8C,D,F,G1–G3). For platelet loss and sC5b-9, slightly higher values were observed for the setups with dialysate (Figure 8B,E); however, these differences were in a much lower magnitude to the observed differences between different dialyzer membranes (Figure 1, Figure 2, Figure 3, Figure 4, Figure 5, Figure 6 and Figure 7).

#### 2.5.2. Investigation of Thrombogenicity Parameters in Experiments with Calcium and Magnesium Supplementation

**Figure 9:**
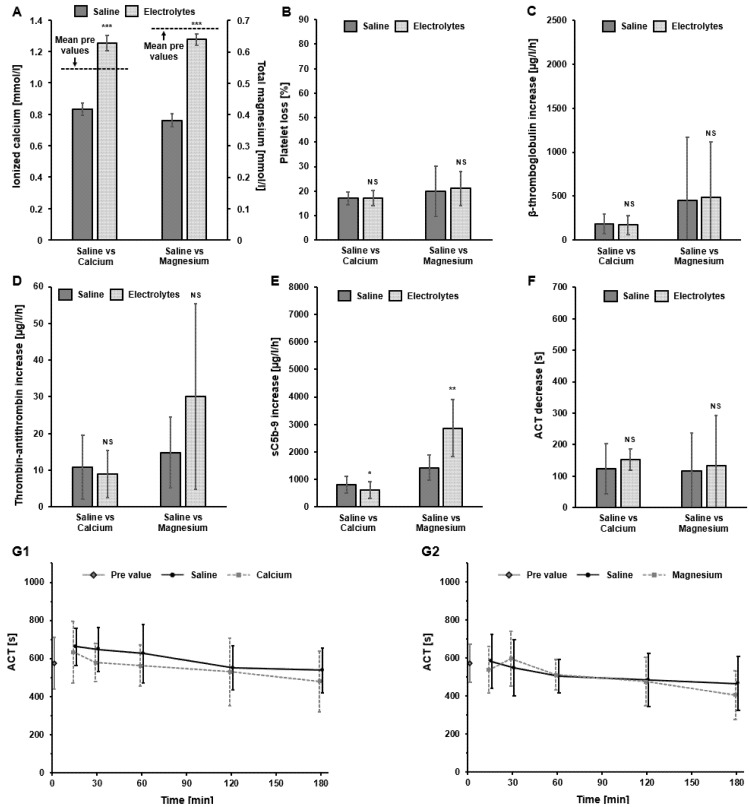
Investigation of thrombogenicity parameters in recirculation experiments with the FX CorAL dialyzer, comparing saline vs. calcium (1.50 mmol/L CaCl_2_) or magnesium (0.50 mmol/L MgCl_2_) supplementation. (**A**) Mean concentration of ionized calcium (left) and total magnesium (right). (**B**) Mean platelet loss. (**C**) Mean β-thromboglobulin (β-TG) increase over time. (**D**) Mean thrombin–antithrombin III complex (TAT) increase over time. (**E**) Mean complement factor sC5b-9 increase over time. (**F**) Mean activated clotting time (ACT) decrease between 15 and 180 min. (**G1**,**G2**) ACT over time for saline vs. calcium supplementation (**G1**) and saline vs. magnesium supplementation (**G2**). Measurements and data presentations are according to Figure 1, Figure 2, Figure 3, Figure 4, Figure 5, Figure 6 and Figure 7, respectively. * *p* < 0.05, ** *p* < 0.01, *** *p* < 0.001, NS: Not significant.

To further investigate any impact of specific electrolytes on thrombogenicity parameters, the saline solution was supplemented with calcium (1.50 mmol/L CaCl_2_) or magnesium (0.50 mmol/L MgCl_2_) in the recirculation setup with the FX CorAL dialyzer. The results of this investigation are summarized in Figure 9.

Supplementation of calcium and magnesium into the saline priming solution significantly increased the ionized calcium and total magnesium levels in the blood, respectively (Figure 9A). When comparing the impact of electrolyte supplementation vs. saline on the investigated thrombogenicity parameters, no significant differences were observed for all markers (Figure 9B–D,F,G1,G2), except for sC5b-9 (Figure 9E). Calcium supplementation significantly decreased complement activation, whereas magnesium supplementation significantly increased complement activation (Figure 9E).

## 3. Discussion

In the present study, we investigated thrombogenicity of different dialyzers in an in vitro test setup. In line with ISO-10993-4, several markers for platelet activation, plasmatic coagulation, and complement activation have been tested, and the influencing factors of these markers were investigated. The results of this study show that the dialyzer has a very strong impact on thrombogenicity. The novel FX CorAL dialyzer with the more hydrophilic membrane showed the lowest platelet and complement activation, supporting the positive impact of hydrophilic membrane modification on dialyzer hemocompatibility.

Patient safety is the most important consideration during the development of a new dialyzer. New dialyzers have to be tested thoroughly before their introduction to the market and their application to dialysis patients. The investigation of dialyzer thrombogenicity is a central part of such testing and must be carried out in line with the current standards [30]. Moreover, such testing has to be conducted in a way that does not lead to artificial results, which may lead to a misinterpretation of dialyzer hemocompatibility.

Several factors may influence in vitro thrombogenicity testing and the impact should be reduced as much as possible. For example, blood samples have a strong variability depending on the blood donors [31,32,33,34]. While using blood from dialysis patients for the extensive in vitro testing during dialyzer development is not feasible, and no standardized method currently exists to mimic uremic blood, the use of human blood from healthy donors is preferable to animal blood, as it provides a more meaningful assessment of blood cell activation. Notably, uremic blood may respond differentially to healthy blood, as uremic conditions can alter protein conformation, adsorption profiles and cellular activation [35,36,37,38]. Previously, we reported that human monocytes undergo reprogramming when incubated with uremic serum, promoting differentiation towards the proinflammatory intermediate monocyte subset [35]. Uremic toxins also impact platelet activation and function, exhibiting both inhibitory and stimulatory effects [36]. Even among healthy donors, baseline levels of the investigated parameters and immunological responses to the dialyzer membrane may differ. Therefore, the proposed setup in this study eliminates this variability by using the same blood in a head-to-head comparison of the new dialyzer and an established reference dialyzer. Moreover, the limited volume of blood available for in vitro testing and the consequently increased tendency towards coagulation necessitate the use of higher anticoagulation doses as compared to clinical settings to prevent fiber blocking and premature testing terminations. Accordingly, the ACT values in the present experiments were higher than those typically observed in dialysis patients [39].

Also, the usage of saline or dialysate for priming and filling of the dialysate side may influence the results by modulating the electrolyte composition in the blood. Of note, using dialysate flow in such an in vitro setup is not feasible, as the limited blood volume would lead to a much stronger removal of small and middle-sized molecules compared to a real dialysis treatment. Nonetheless, the usage of dialysate instead of saline in such an in vitro setup may be beneficial for certain parameters such as complement activation, especially in comparisons of dialyzers with big differences in the volumes of the blood and dialysate compartments. Such different dialyzer designs may have an impact on the electrolyte composition in the recirculating blood, such as on calcium and magnesium, which are well known to modulate thrombogenicity [40,41,42,43,44]. In line with this, in the present study, the use of dialysate instead of saline to achieve physiological electrolyte concentrations resulted in a slight increase in platelet loss and complement activation. Detailed analyses of electrolyte supplementation revealed that calcium attenuates complement activation, whereas magnesium enhances it. Also, differences in membrane surface areas among the investigated dialyzers should be kept as small as possible. Moreover, dialyzers with smaller membrane surface areas are generally preferred, due to the limited blood volume available for in vitro testing. The selection of membrane sizes should also take into account that different hemocompatibility markers may have specific detection limits, and membrane surface areas that are too small or too large may result in invalid measurements if values fall below or exceed these limits.

However, the results of this study show that the dialyzer membrane has the strongest effect on thrombogenicity. Therefore, in vitro testing of dialyzer membranes, which have strong adsorptive properties, such as the AN69 ST membrane, have to be performed and interpreted with care. The low levels of certain activation markers may be a result of adsorption of these factors to the membrane and not by a seemingly low activation. Therefore, when comparing a new dialyzer to a dialyzer with a different membrane material, such an impact on the results has to be considered. Moreover, the use of dialysate instead of saline as a priming solution may be beneficial in such comparisons. Besides membrane material, the geometry of the fiber, particularly the fiber length, inner diameter and membrane surface area, may also be relevant in this context, as these parameters influence hemodynamic conditions such as pressure drop and shear stress, which in turn may affect hemocompatibility [45,46]. Moreover, the outer dialyzer design may also affect the hemodynamic conditions, especially the design of the flanges and the connection to the tubing system. The designs with the lateral blood ports may be advantageous, due to more homogenous blood flow distribution within the flange [47].

In the present study, the novel FX CorAL dialyzer was compared to six other polysulfone- or polyethersulfone-based dialyzers. In most of the head-to-head comparisons, the FX CorAL dialyzer showed the lowest platelet and complement activation, indicating that the membrane material has the largest impact on hemocompatibility. Moreover, the platelet results correlated with the ACT data, showing a slight increase at the start of the recirculation. During the recirculation, the ACT decreased for all dialyzers pointing towards an ongoing coagulation process; notably, the ACT decrease was lowest for the FX CorAL dialyzer. However, plasmatic coagulation, as determined by TAT, did not show any differences between the investigated dialyzers.

The positive hemocompatibility effects by the FX CorAL can be attributed to its new membrane [47]. All investigated polysulfone- and polyethersulfone-based dialyzers in this study use polyvinylpyrrolidone (PVP) as a co-polymer, which is a very hydrophilic agent. Increased membrane hydrophilicity is associated with lower protein adsorption to the membrane, better hemocompatibility, and more stable performance over time [16,22,23,24,25,26,27,48]. The membrane of the FX CorAL dialyzer was modified by specifically increasing the PVP concentration on the blood-contacting surface of the hollow fiber [17]. Moreover, in contrast to autoclave steam or gamma sterilization, the INLINE-steam sterilization process utilized for the FX CorAL dialyzer was shown to prevent PVP elution from the membrane; thus, the positive PVP effects are maintained over the treatment duration time [17].

Three clinical studies evaluated the hemocompatibility profile of the FX CorAL dialyzer (FX CorAL 600 [1.6 m^2^ membrane surface]) and of comparable dialyzers, which were also investigated in the present study (FX CorDiax 600 [1.6 m^2^] and xevonta Hi 15 [1.5 m^2^]), as well as of additional dialyzers (FX 600 [Fresenius Medical Care; 1.6 m^2^], Sureflux 17UX [Nipro; 1.7 m^2^], Polyflux 170H [Baxter; 1.7 m^2^]) [20,49,50]. The markers of platelet activation (platelet counts and β-TG), coagulation (TAT), and complement activation (sC5b-9) were also investigated in these clinical studies with 179 patients in total. These results confirmed that the FX CorAL dialyzer induces lower platelet and complement activation while showing comparable plasmatic coagulation. Of note, no clinical study evaluated the FX CorAL against the other investigated dialyzers from the present study with regard to thrombogenicity (Optiflux, ELISIO, Revaclear, Theranova, Nephral). However, previous in vitro studies with the FX CorAL 600, which has a larger membrane surface (1.6 m^2^) than the dialyzer used in the present study (FX CorAL 60; 1.4 m^2^), confirmed the current findings of lower platelet and complement activation compared to the FX CorDiax 600 (1.6 m^2^), xevonta Hi 15 (1.5 m^2^), ELISIO 15H (1.5 m^2^) and Theranova 400 (1.7 m^2^) dialyzers, thereby suggesting that the smaller membrane surface of the FX CorAL 60 in current comparisons is unlikely to have confounded the present results. For the Nephral dialyzer, no direct clinical comparison with the FX CorAL has been conducted to date and limited data in terms of hemocompatibility are available in general. Few case reports [51,52] described lower adverse effects or platelet loss in patients who experienced reactions with various synthetic membranes before. However, the proposed mechanism of lower complement activation due to the adsorption effects of the complement factors was not supported by direct measurements [51], and our present in vitro findings do not support generally lower complement activation by the Nephral dialyzer.

Improvements in dialyzer hemocompatibility may contribute to better short-term and long-term clinical outcomes. Four clinical studies investigated the safety profile of the FX CorAL dialyzer and showed a high level of safety, with no adverse device effects attributed to hemocompatibility-related complications across a total of 1389 treatments [20,49,50,53]. Ongoing post-market surveillance of the FX CorAL dialyzer provides continuous insights into its safety profile, supporting its use in a broad range of patient populations. These data will also help to clarify whether the FX CorAL can serve as a suitable alternative for patients experiencing hypersensitivity reactions with synthetic membranes who would otherwise be switched to dialyzers with other membrane types, such as cellulose-based membranes. Moreover, longitudinal data are needed to determine whether the improvements in hemocompatibility will translate into long-term clinical benefits, such as reduced cardiovascular events.

In conclusion, the present study proposes guidance on in vitro testing of dialyzer thrombogenicity, in line with ISO-10993-4, and helps to reduce current heterogeneity among in vitro hemocompatibility testing. Using the example of the novel FX CorAL dialyzer, the study investigated critical parameters of platelet activation, coagulation and complement activation and analyzed factors which may influence in vitro testing. The present results are in line with previous in vitro studies and clinical trials, showing that the FX CorAL dialyzer has a favorable hemocompatibility profile.

## 4. Materials and Methods

### 4.1. Investigated Dialyzers

The novel polysulfone-based FX CorAL was the test device in this study and was compared to three further polysulfone-, three polyethersulfone- and one AN69 ST-based dialyzers. The sizes of the dialyzers were selected based on availability and to keep differences in the membrane surface area as small as possible (1.4 m^2^–1.8 m^2^). Further information regarding the investigated dialyzers, including the membrane material, sterilization method, blood priming volume, pressure drop and fiber inner diameter, are provided in Table 1. Moreover, regarding the outer dialyzer designs, the FX CorAL and FX CorDiax feature a flange design with horizontal blood inlet and outlet ports, whereas the other dialyzers have vertical ports.

### 4.2. Blood Collection

For the in vitro experiments, apparently healthy volunteers were recruited. The donors were not taking medications that were known to affect platelets or the coagulation system and provided written informed consent. In total, 450 mL of whole blood was drawn from a vein at the antecubital fossa, using a 17 G dialysis needle (Fresenius Medical Care, Bad Homburg, Germany), into a heparin prefilled bag (750 IU [Ratiopharm GmbH, Ulm, Germany] in 50 mL saline solution [Fresenius Medical Care]; final concentration: 1.5 IU/mL). Due to the lower blood volume and subsequently higher degree of coagulation activation in the in vitro test setup, a higher anticoagulation dose was used than in a clinical situation, to allow for an efficient in vitro test run, without testing failures due to increased coagulation and subsequent fiber blocking. All experiments were initiated within 30 min of blood collection.

### 4.3. Blood Recirculation Setup

**Figure 10:**
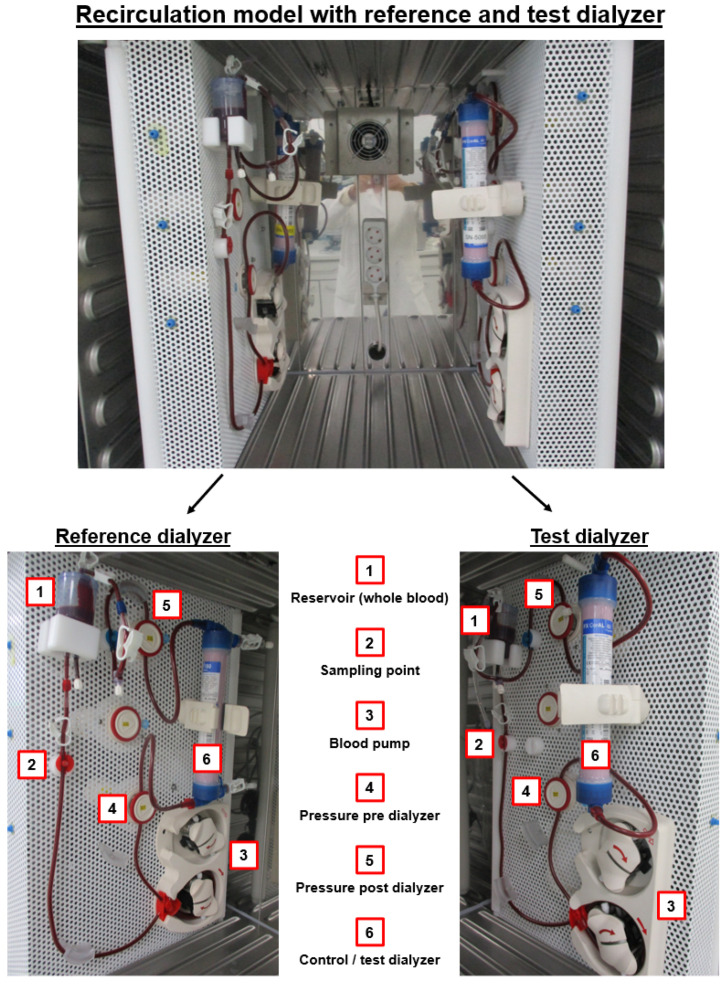
In vitro blood recirculation setup. The test dialyzer and the reference dialyzers were investigated in the same benchtop setup, using blood from the same donor. At different time points of blood recirculation, blood samples were taken for the measurements of thrombogenicity markers. The tubing system was a tailor-made assembly manufactured by Fresenius Medical Care and did not contain any anticoagulant coatings or other surface modifications. It had a total length of 2135 mm, a total volume of 43 mL, and an inner and outer diameter of 4.3 and 6.8 mm, respectively, except for the pump segment, which had an inner diameter of 8.0 mm and an outer diameter of 12.2 mm (total inner surface area of the tubing system: ~0.03 m^2^). The blood pump was a peristaltic pump that was identical to the model implemented in the 5008 hemodialysis machine from Fresenius Medical Care. Mean pre-dialyzer pressure vales across the experiments were: 99.4–105.0 mmHg (FX CorAL), 104.3 mmHg (FX CorDiax), 94.2 mmHg (Optiflux), 88.5 mmHg (xevonta), 89.8 mmHg (ELISO), 80.3 mmHg (Revaclear), 82.2 mmHg (Theranova) and 93.7 mmHg (Nephral).

To evaluate the thrombogenicity of a new dialyzer under controlled and standardized conditions, two identical circuits in the same incubator (37 °C; Memmert, Schwabach, Germany) were used for the reference dialyzer and the respective test dialyzer (Figure 10). The reference dialyzer was an established product on the market and was used as a reference to which the new test dialyzer was compared. After pre-rinsing the two recirculation systems with saline solution for ~30 min, they were filled with blood from the same pool (i.e., ~220 mL per system). The dialysate compartment remained closed during the complete test run. For the additional comparison experiments with dialysate instead of saline solution, the following bicarbonate dialysis solutions were used: (1) AC-F 211.0 (Na^+^: 138.00 mmol/L, K^+^: 2.00 mmol/L, Ca^2+^: 1.00 mmol/L, Mg^2+^: 0.50 mmol/L, Cl^−^: 108.00 mmol/L, HCO_3_^−^: 32.00 mmol/L, acetate: 3.00 mmol/L; glucose: 1.00 g/L), (2) AC-F 211.5 (Na^+^: 138.00 mmol/L, K^+^: 2.00 mmol/L, Ca^2+^: 1.50 mmol/L, Mg^2+^: 0.50 mmol/L, Cl^−^: 109.00 mmol/L, HCO_3_^−^: 32.00 mmol/L, acetate: 3.00 mmol/L; glucose: 1.00 g/L), and (3) multi*Plus* (Na^+^: 140.00 mmol/L, K^+^: 2.00 mmol/L, Ca^2+^: 1.50 mmol/L, Mg^2+^: 0.75 mmol/L, Cl^−^: 109.70 mmol/L, HCO_3_^−^: 35.00 mmol/L, inorganic phosphate: 1.00 mmol/L; glucose: 1.00 g/L) (all Fresenius Medical Care). Moreover, additional experiments were performed to specifically increase calcium or magnesium concentrations in the setup. Therefore, the saline solution was supplemented with CaCl_2_ (1.50 mmol/L) or MgCl_2_ (0.50 mmol/L).

The blood flow rate was set to 200 mL/min, and the blood was recirculated for 180 min. At distinct time points (15, 30, 60, 120 and 180 min), blood samples were taken at the sampling point (Figure 10), using a 20 G needle connected to anticoagulant prefilled blood collection tubes (Sarstedt, Nürnbrecht, Germany). The total volume of blood per connection time point was 10 mL (1.6 mL K3 EDTA [blood cell count], 1.2 mL K3 EDTA [sC5b-9], 1.4 mL citrate 9NC [TAT], 2.9 mL CTAD [β-TG], 2 × 1.2 mL lithium heparin [ionized calcium and total magnesium] and 0.5 mL collected in a syringe for the ACT measurement). Moreover, a pre-treatment sample was directly taken from the blood pool bag and used for the measurement of the pre values (0 min).

### 4.4. Determination of Thrombogenicity Markers

To evaluate (1) platelet activation, (2) plasmatic coagulation, and (3) complement activation, the following markers were measured at the respective time points (0 min to 180 min): (1) platelet counts and β-thromboglobulin [βTG], (2) Thrombin–antithrombin III complex [TAT], and (3) complement factor sC5b-9. Additionally, for control purposes, the activated clotting time (ACT) and ionized calcium and total magnesium concentrations were determined. The respective methods and kits are given in Table 2.

### 4.5. Evaluation of Thrombogenicity Markers

For the evaluation of platelet loss, the respective counts were first corrected for the hematocrit (corrected platelet count [t] = platelet count [t] × hematocrit [start]/hematocrit [t]). Then, the area under the curve (AUC) was calculated in the time up to 60 min, to capture the typical platelet drop in this timeframe. The percentage platelet drop was calculated based on the AUC per min, relative to the pre-treatment samples (0 min). For β-TG, TAT and sC5b-9, the AUC was determined in the time up to 180 min and corrected for the respective pre values. The concentration increase per hour was then calculated for the three parameters. For ACT, the clotting time was also determined at each time point and the difference between 15 min and 180 min was compared between the reference and test dialyzers. For the ionized calcium and total magnesium, the mean concentrations across all measurements between 15 min and 180 min were compared between the different dialyzers.

All data are presented as the mean ± standard deviation (SD) of six independent experiments. The pairwise comparisons were statistically analyzed using Student’s *t*-test. *p*-values < 0.05 were considered statistically significant (* *p* <0.05, ** *p* < 0.01, *** *p* < 0.001).

### 4.6. Determination of Platelet Adsorption by Fluorescence Microscopy

To visualize platelet adsorption to the different membranes, fluorescence microscopy imaging was performed. Therefore, the dialyzers were first exposed to blood recirculation as described in Section 4.3. According to the determination of platelet loss (Section 4.5), blood was recirculated for 60 min to allow for a comparison between fluorescence microscopy and hematology. After blood recirculation, the dialyzers were rinsed with one liter of saline solution. The dialyzers were immediately opened, and random fibers were removed for fluorescence labeling of attached platelets. Then, the fibers were attached to microscope slides using double sided tape, cut open, and flattened on the slides. No further fixation step was performed to avoid structural damage of the membranes and the subsequent loss of platelets from the membrane. FITC labeled antibodies that target CD41a (BD Biosciences, Franklin Lakes, NJ, USA; in tris buffered saline, pH 7.4, with 2% fetal bovine serum), corresponding to the platelet activation marker glycoprotein IIb/IIIa complex, were used to stain the adhered platelets on the inner membrane surface. The samples were incubated in a dark and humidified chamber overnight. Afterwards, the antibody solution was removed, and the slides were washed four times with 200 mL tris buffered saline (pH 7.4) with 1% Tween-20, swirling gently for 30 s each time, to remove extraneous antibodies that were not attached to the platelets on the dialyzer fibers. Labeled platelets were visualized using a BX31 microscope equipped with a DP73 camera (Olympus, Center Valley, PA, USA), using indirect illumination. The fluorescence detection filters utilized a 450–490 nm excitation filter, a 510 nm dichroic mirror, and a bypass filter of 520–560 nm band-width. CellSense software, version 4.1.16 (Olympus) was used for processing. The images were visually inspected.

## Figures and Tables

**Table 1:** Investigated dialyzers in the present study.

Dialyzer	Manufacturer	Membrane Name	Membrane Material	Sterilization	Blood Priming Volume [mL]	Pressure Drop [mmHg]	Fiber Inner Diameter [µm]	Membrane Surface [m^2^]
FX CorAL 60	Fresenius Medical Care	Helixone *hydro*	Polysulfone, PVP	INLINE steam	80	86	185	1.4
FX CorDiax 60	Fresenius Medical Care	Helixone *plus*	Polysulfone, PVP	INLINE steam	80	86	185	1.4
Optiflux F160NRe	Fresenius Medical Care	Advanced Fresenius Polysulfone^®^	Polysulfone, PVP	Electron beam	87	89	185	1.5
xevonta^®^ Hi 15	B. Braun	Amembris Polysulfone	Polysulfone, PVP	Gamma	90	128	195	1.5
ELISIO^TM^-17H	Nipro	Polynephron^TM^	Polyethersulfone, PVP	Gamma	105	67 ^#^	200	1.7
Revaclear 400	Baxter/Vantive	Poracton	Polyarylethersulfone, PVP	Steam	93	N/A	190	1.8
Theranova 400	Baxter/Vantive	N/A (MCO)	Polyarylethersulfone, PVP	Steam	91	≤130	180	1.7
Nephral ST 400	Baxter/Vantive	N/A	AN69 ST *	Gamma	100	84	210	1.65

PVP: polyvinylpyrrolidone; N/A: not available; MCO: medium cut-off; ^#^ determined at 200 mL/min, the other dialyzers at 300 mL/min; and * acrylonitrile sodium methallyl sulfonate blend.

**Table 2:** Used methods and kits for the evaluation of thrombogenicity markers.

Thrombogenicity Parameter	Marker	Method/Kit
Platelet activation	Platelet counts (including other hematology markers *)	Automatic hematology analyzer (XN-1000 Pure analyzer, Sysmex, Kobe, Japan)
	β-thromboglobulin [β-TG]	ELISA (Asserachrom ß TG, Diagnostica Stago, Asnières-sur-Seine, France)
Plasmatic coagulation	Thrombin–antithrombin III complex [TAT]	ELISA (Enzygnost TAT micro, Siemens Healthineers AG, Forchheim, Germany)
Complement activation	Complement factor sC5b-9	ELISA (MicroVue™ sC5b-9 Plus EIA, QuidelOrtho, San Diego, CA, USA)
Additional clotting-related parameter	Activated clotting time (ACT)	Automatic coagulation timer system (low range activated clotting time, ACT Plus^®^, Medtronic GmbH, Meerbusch, Germany)
	Ionized calcium	Blood gas analyzer (ABL80 Flex Basic, Radiometer GmbH, Krefeld, Germany)
	Total magnesium	Clinical–chemical analyzer (Cobas^®^ c303 Analyzer, Roche Diagnostics AG, Rotkreuz, Switzerland)

* Additional hematology markers: leukocyte counts, red blood cell counts, hematocrit, hemoglobin; and ELISA: enzyme-linked immunosorbent assay.

## Data Availability

Data are contained within the article.

## Abbreviations

The following abbreviations are used in this manuscript:

ACT	Activated clotting time
AN69 ST	Acrylonitrile sodium methallyl sulfonate blend
AUC	Area under the curve
β-TG	β-thromboglobulin
F1.2	Prothrombin fragment 1.2
FITC	Fluorescein isothiocyanate
FPA	Fibrinopeptide A
MCO	Medium cut-off
PF4	Platelet factor 4
PTT	Partial thromboplastin time
PVP	Polyvinylpyrrolidone
sC5b-9	Complement factor sC5b-9
SD	Standard deviation
TAT	Thrombin–antithrombin III complex
TXB2	Thromboxane B2
